# Microscopic and molecular epidemiology of gastrointestinal nematodes in dairy and beef cattle in Pak Chong district, Nakhon Ratchasima province, Thailand

**DOI:** 10.14202/vetworld.2024.1035-1043

**Published:** 2024-05-09

**Authors:** Chompunoot Wangboon, Pongsakorn Martviset, Mantana Jamklang, Sirilak Chumkiew, Watsana Penkhrue, Sainamthip Rangdist, Ruttiroj Jirojwong, Wansika Phadungsil, Pathanin Chantree, Rudi Grams, Dawid Krenc, Pantip Piyatadsananon, Amornrat Geadkaew-Krenc

**Affiliations:** 1 School of Preclinical Sciences, Institute of Science, Suranaree University of Technology, Muang, Nakhon Ratchasima 30000, Thailand; 2 Department of Preclinical Science, Faculty of Medicine, Thammasat University, Khlong Luang, Pathum Thani 12120, Thailand; 3 School of Biology, Institute of Science, Suranaree University of Technology, Muang, Nakhon Ratchasima 30000, Thailand; 4 Department of Livestock Development, Bureau of Veterinary Biologics, Pak Chong, Nakhon Ratchasima, 30130, Thailand; 5 Graduate Program in Biomedical Sciences, Faculty of Allied Health Sciences, Thammasat University, Khlong Luang, Pathum Thani 12120, Thailand; 6 Chulabhorn International College of Medicine, Thammasat University, Khlong Luang, Pathum Thani 12120, Thailand; 7 School of Geoinformatics, Institute of Science, Suranaree University of Technology, Nakhon Ratchasima 30000, Thailand

**Keywords:** beef cattle, dairy cattle, gastrointestinal nematode, strongyle nematodes

## Abstract

**Background and Aim:**

Gastrointestinal (GI) nematode infection remains an important problem in livestock, particularly cattle. The infection may lead to serious health complications and affect animal products. The objective of this study was to investigate GI nematode infection and its associated risk factors in dairy and beef cattle farmed in Pak Chong District of Nakhon Ratchasima Province, northeast Thailand.

**Materials and Methods:**

Fecal specimens were collected from 101 dairy cattle and 100 beef cattle. Formalin-ethyl acetate concentration techniques were used to process the samples and the samples were observed under a light microscope. Samples were subjected to molecular identification of specific genera using conventional polymerase chain reaction and DNA sequencing.

**Results:**

The overall prevalence of GI nematode infection was 33.3%. The strongyle nematode was the most significant GI nematode in this area with a prevalence of 28.4%. The prevalence of strongyle nematodes was 58.0% in beef cattle and only 7.9% in dairy cattle. *Trichuris* spp. was another nematode found in both types of cattle with an overall prevalence of 5.0% with 9.0% in beef cattle and 1.0% in dairy cattle. The results of the epidemiological study indicate that the age of cattle, food, water sources, farming system, and housing floor are the most important risk factors. Among the strongyle nematodes, *Ostertagia* spp. was the most prevalent (82.0%), followed by *Haemonchus* spp. (62.3%) and *Trichostrongylus* spp. (8.2%), respectively.

**Conclusion:**

Infection with GI nematodes still exists in this area, particularly in beef cattle. Our reported data may benefit local parasitic control policies in the future.

## Introduction

Parasitic infection in ruminants, especially in economically important animals, is a crucial concern due to several effects on animal health and the quality of animal products [1]. Gastrointestinal nematodes (GINs) are the major infective organisms in ruminants [2, 3]. GIN infections cause a wide range of symptoms and complications, from mild to severe, depending on the species of parasite, their invasive or non-invasive pathogenesis, and parasite number [4, 5]. Strongyle nematodes belong to the family *Strongylidae* and comprise several genera such as *Trichostrongylus* (TS), *Haemonchus*, *Ostertagia*, *Cooperia*, *Nematodirus*, and *Oesophagostomum* [6]. *Trichuris* spp., *Strongyloides* spp., *Toxocara* spp., and *Capillaria* spp. are also frequently found nematodes with various parasitic numbers [3, 7]. Even when anthelmintics are administered, the number of cases is not much reduced due to misuse and overuse of the drugs, leading to drug resistance [3]. Thailand is a tropical country located in Southeast Asia, where many agricultural industries exist. Not only rice, rubber, and sugar, but also meat products are important [8]. Beef and dairy cattle are economically important animals in the country, with more than 1.0 million heads in 2015 [8, 9]. GIN infections affect the cattle industry by lowering product quality and quantity; moreover, they affect animal health by causing death in cases of heavy infection [1]. Jittapalapong *et al*. [10] reported that strongyle nematodes shared the greatest percentage among helminths with 6.1% countrywide prevalence in cattle and the highest prevalence in the southern region (19.3%) in Thailand. Yuwajita *et al*. reported that the prevalence of strongyles was 13.7% in cows farmed in Udon Thani [11]. Moreover, recent prevalence data for strongyles have been reported for Kanchanaburi province, western Thailand, 28.7% [12] and Kalasin province, Northeast Thailand, 84.2% [13].

Pak Chong district of Nakhon Ratchasima Province, northeast Thailand, is one of the biggest beef and dairy cattle farming areas in Thailand, where more than 97,000 cattle were farmed in 2021 (Department of Livestock Development, Ministry of Agriculture and Cooperatives of Thailand, 2021). However, there have been no reports of strongyle infection in this area.

Therefore, this study aims to evaluate the prevalence of strongyle nematodes by comparing beef and dairy cattle and to demonstrate the genera of infective parasites and associated risk factors in the sampled areas using molecular techniques. The finding of this research is not only in reporting the prevalence of strongyle in beef and dairy cattle but also in the benefit for public policy at the local administration level in Thailand.

## Materials and Methods

### Ethical approval and Informed consent

The Institutional Animal Care and Use Committee of Thammasat University approved the animal ethics of this study (project reference no. 025/2622). The Thammasat University Institutional Biosafety Committee approved the biosafety protocol (reference no. 052/2565).Verbal consent was obtained from each animal owner.

### Study period and locations

**Figure-1:**
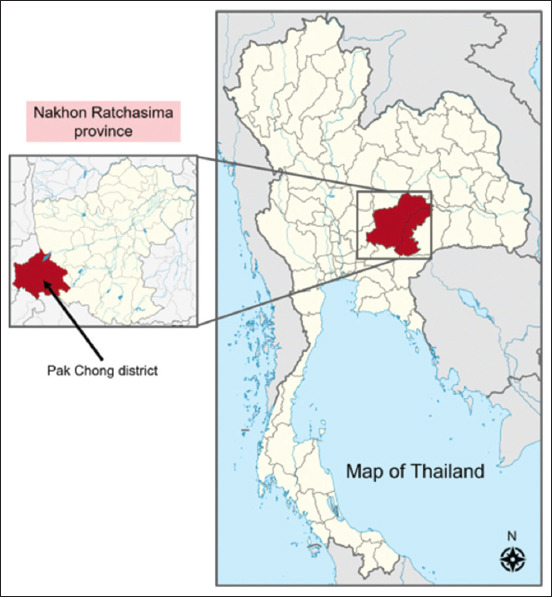
Map of Pak Chong district, Nakhon Ratchasima province, Thailand [Source: https://commons.m.wikimedia.org/wiki/Atlas_of_Thailand].

This study was conducted from October to December 2020 in Pak Chong district, Nakhon Ratchasima province, northeast Thailand. Farms were randomly selected from all 12 sub-districts of Pak Chong district, as shown in Figure-1. This study included 25 selected farms, 101 dairy cattle, and 100 beef cattle (Table-1). Epidemiological data, including age, food (natural grasses or mixed foods), water source (natural pond, tap, or groundwater), housing floor (soiled or mixed floor), and farming system (open or closed systems) were collected from all cattle.

**Table 1:** Number of fecal specimens collected from all 12 sub-districts in Pak Chong district.

Sub-district (“Tambon”)	Dairy cattle	Beef cattle	Total number of fecal samples	Number of selected farms
Chan Thuck	10	10	20	4
Pak Chong	8	12	20	2
Nong Sa Rai	10	10	20	3
Wang Sai	10	8	18	1
Klangdong	6	6	12	1
Nong Nam Dang	7	9	16	2
Kanong Pra	10	5	15	2
Phrayayen	6	6	12	2
Moo Si	6	9	15	1
Pong Ta Long	10	5	15	1
Wang Ka Ta	10	10	20	2
Klong Muang	8	10	18	4
Total	101	100	201	25

### Fecal collection and microscopic examination

Fecal specimens were collected by enema from 201 cattle (100 beef and 101 dairy cattle). Samples were placed in sterile containers in a chilled insulated box and transported to the laboratory within 1–2 h. The samples were processed immediately after arriving in the laboratory at the Institute of Science, Suranaree University of Technology, Muang district, Nakhon Ratchasima province. Fresh specimens were investigated for helminth infection using the formalin-ethyl acetate concentration technique (FECT) [14–17] and observed under a light microscope. Helminth eggs were identified, and GI nematode eggs were quantitatively reported as eggs per gram. Microscopic-positive samples with GI nematode infection were subjected to genomic DNA extraction.

### Genomic DNA extraction

Genomic DNA was extracted using the QIAamp^®^ DNA Mini Kit (QIAGEN, Hilden, Germany) following the manufacturer’s instructions from microscopic-positive specimens with GI nematode eggs. The final filtrate contained 50 μL of the eluted DNA solution. A NanoDrop spectrophotometer (Thermo Fisher Scientific, Wilmington, DE, USA) was used to determine the concentration and purity of the extracted DNA samples. DNA was stored at –20°C until processing as a template for polymerase chain reaction (PCR).

### PCR amplification

The morphological characteristics of strongyle nematode eggs are similar; therefore, the detection of the three major parasites that are commonly found in cattle, including *Ostertagia ostertagi* (OS), *Haemonchus contortus* (HC), and TS. [18–20], was performed by PCR amplification using species-specific primers (Table-2) [21, 22]. PCR reactions were performed in a total volume of 25 μL as previously described by Martviset *et al*. [23] using GoTaq® Hot Start Colorless Master Mix (Promega, WI, USA) in a thermal cycler (BIORAD T100 thermal Cycler, Singapore). The amplification steps consisted of 1 cycle of initial denaturation at 95°C for 2 min, followed by 30 cycles of 95°C for 30 s, 55–65°C for 45 s, and 72°C for 45 s, and a final extension at 72°C for 5 min. The positive controls for OS and TS were synthetic DNA fragments (GeneArt™ Strings™, ThermoFisher Scientific, Regensburg, Germany) and isolated adult-stage genomic DNA for (HC). The PCR products were size-separated on 2% agarose gels containing ViSafe Green Gel Stain (Vivantis, Shah Alam, Malaysia) using 1× TBE buffer at 100 V for 35 min. The gels were then photographed under ultraviolet light (Gel Doc Vilber, France).

**Table 2:** Forward and reverse primers used for genus identification of strongyle nematodes.

Nematode	Target gene	Oligonucleotide sequence (5’⟶3’)	Size (bp)	Reference
*Haemonchus contortus*	ITS1	fw: GTATCACCCCGTTTAAAGCTC	345	[21]
		rv: GATCCATCGCTGAAGCTAATC		
*Ostertagia ostertagi*	ITS1	Fw: TGGGAGTATCACCCCCGTTA	73	[22]
		Rv: TCGCCACTCATGAACGACTC		
*Trichostrongylus* spp.	ITS-2	Fw: TGTTCCTGTATGATGTGAACGTG	128	[22]
		Rv: CGCCTGAGTTCAGGTTGC		

### Molecular analysis of strongyle nematodes

The PCR products of each specific primer pair were purified and sent for DNA sequencing (Bionics Co., Ltd., Seoul, South Korea). Sequence analysis was performed using EMBOSS (EMBOSS programs: https://www.ebi.ac.uk/services) < EMBL-EBII. The evolutionary phylogenetic tree was generated by MEGA11 (https://www.megasoftware.net) using the maximum likelihood method with 1000 bootstrap replications [24, 25].

### Statistical analysis

Data were analyzed using the Statistical Package for the Social Sciences (SPSS) for Windows, version 28.0 (SPSS, Chicago, IL, USA) and GraphPad Prism version 9 (GraphPad Software, Inc., Boston, MA, USA). Univariate and multivariate logistic regression analyses were performed to determine the associated factors, including age, food, water source, housing floor, and farming system. The magnitudes of the associations obtained from univariate and multivariate analyses are represented as odds ratios (ORs) with the corresponding 95% confidence interval (CI). Fisher’s exact test and Pearson’s Chi-square test were also used on the categorical data. The prevalence of strongyle nematodes is described in percentages. A p < 0.05 was considered statistically significant.

## Results

### Microscopic examination for infection rate and eggs of gastrointestinal (GI) helminths in cattle

**Figure-2:**
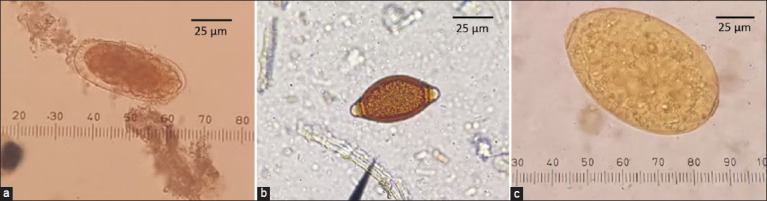
Parasite eggs found in the formalin-ethyl acetate concentration techniques sediments at 400× magnification. (a) Strongyle egg, (b) *Trichuris* spp. egg, (c) *Fasciola* spp. egg.

**Figure-3:**
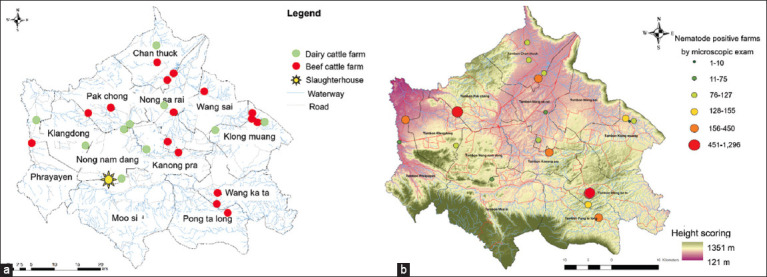
(a) Geographical map of dairy cattle and beef cattle farms in this study. (b) The intensity of nematode infection (eggs per gram) is shown for the nematode-positive farms.

In 2020, fecal specimens were collected from 10 dairy and 15 beef cattle farms from all 12 sub-districts in Pak Chong, Nakhon Ratchasima province, Thailand All fecal samples of dairy cattle (n = 101) and beef cattle (n = 100) were analyzed by FECT to observe the GI helminth eggs. The helminth eggs were *Strongyle* spp., *Trichuris* spp., and *Fasciola* spp. (Figure-2). The GI nematode infection rate was 33.3% (67/201), which was dominant compared to the 6.0% trematode *Fasciola* spp. (12/201), as reported in our previous study by Martviset *et al*. [17]. The studied farms and the farms with microscopic-positive semi-quantitative GI nematode eggs are shown in Figure-3. Eighteen of the 25 farms located in Pak Chong district had cattle infected with GI nematodes.

In dairy cattle, the overall prevalence of GI nematodes was 8.9% (9/101), including 7.9% (8/101) strongyle nematode and 1.0% (1/101) *Trichuris* spp. In contrast, beef cattle had a higher prevalence of GI nematodes, with an overall prevalence of 58.0% (58/100), including 49.0% (49/100) strongyle nematodes and 9.0% (9/100) *Trichuris* spp. Details are shown in Table-3.

**Table 3:** Overall microscopy prevalence of gastrointestinal nematode infections in dairy and beef cattle.

Parasites	Dairy cattle (n = 101)		Beef cattle (n = 100)		Overall (%)
	
	Positive samples	Infection (%)	Positive samples	Infection (%)	
*Trichuris* spp.	1	0.99	9	9	4.98
Strongyle nematode	8	7.92	49	49	28.35
Total	9	8.91	58	58	33.33

### Molecular detection and identification of GI nematodes using PCR

**Figure-4:**
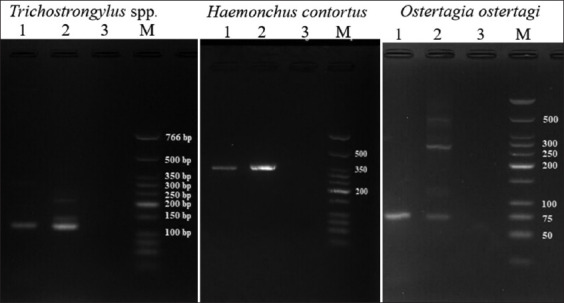
Polymerase chain reaction amplicons from positive specimens. Lanes 1 contained *Trichostrongylus* spp., *Haemonchus* *contortus*, and *Ostertagia ostertagi* DNA. The corresponding positive controls were in lanes 2 and negative controls in lanes 3. DNA markers were in lanes M.

To maximize the detection limit of strongyle nematodes, which are the most important GI nematodes, all positive samples were subjected to PCR amplification with strongyle-specific primer sets. Only 61 out of 67 stool samples could be processed due to the amount of collected stool samples. Simultaneously, strongyle nematode species were assessed using the same reaction. Positive results were determined by agarose gel electrophoresis and later DNA sequencing. Examples of PCR amplicons of all three parasites compared with positive and negative controls are shown in Figure-4.

**Figure-5:**
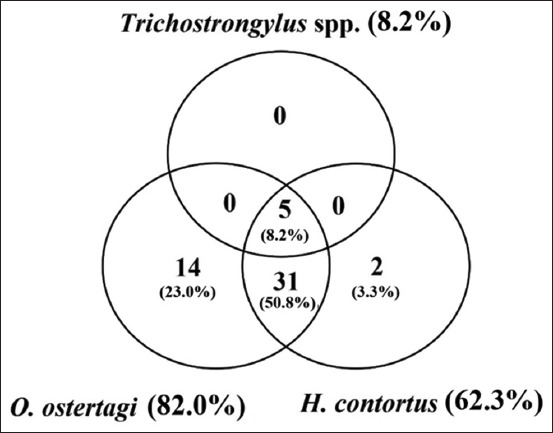
Venn diagram summarizing the infection of strongyle nematode infections.

The highest prevalence among the strongyle nematodes was for OS (82.0%, 50/61), followed by (HC) (62.3%, 38/61) and TS. (8.2%, 5/61). Interestingly, double and multiple infections with strongyle nematodes accounted for 59.0% (36/61), 8.2% (5/61) triple infection, and 50.8% (31/61) double infection with OS and (HC). Figure-5 shows a Venn diagram summarizing the infection with strongyle nematodes.

### Phylogenetic analysis

**Figure-6:**
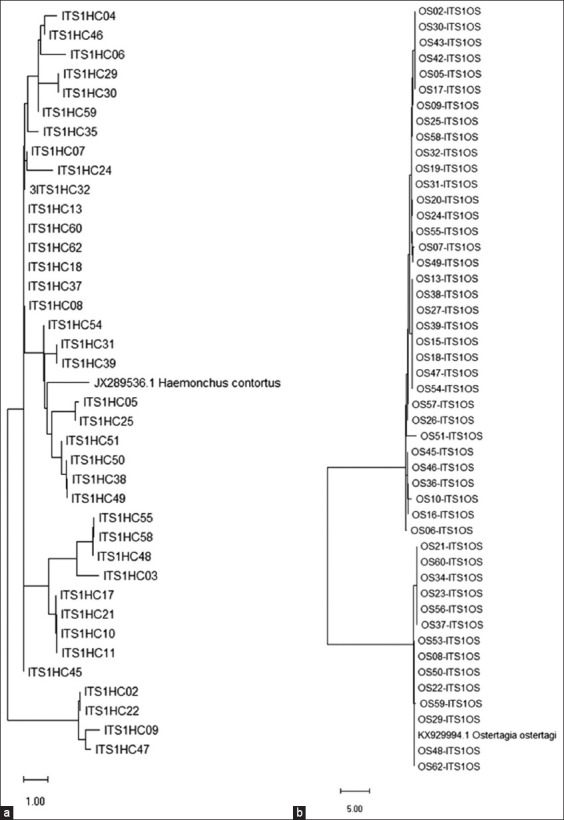
Evolutionary phylogenetic tree of strongyle nematodes found in this study. (a) Haemonchus contortus and (b) Ostertagia ostertagi.

Phylogenetic trees were generated separately from positive samples of HC, OS, and TS, using the sequences of HC ITS1 (JX289536.1), OS ITS1 (KX929994.1), and *Trichostrongylus colubriformis* ITS-2 (KU891930.1) as references. All PCR-positive samples were included in the construction of the phylogenetic tree. The HC tree revealed three distinct groups separated into three major branches. However, when we looked at the scores, they were not very diverse. Most of them were closely related to the reference strain; therefore, it can be concluded that all HC primer-positive samples were HC. On the other hand, the OS tree showed two major branches. A smaller number were closely related to the OS reference strain, whereas most of them were more distantly related. For TS, the number of samples was only five, so the phylogenetic tree could not be generated with confidence. However, the phylogenetic analysis confirmed the microscopic and molecular identification findings. Figure-6 shows the evolutionary phylogenetic trees.

### Epidemiologically associated risk factors

The risk factors associated with GI nematode infections were investigated (Table-4). The age of the cattle was related to GI nematode infection in all cattle. Young cattle demonstrated a higher prevalence than older cattle (p < 0.001). Interestingly, dairy cattle showed the same trend, although statistical analysis could not be performed because some data sets fell to zero. Beef cattle showed a statistically insignificant difference in prevalence among age groups (p = 0.974). In terms of food consumption, natural grasses may pose a risk of infection in both types of cattle compared to mixed foods containing commercially prepared components. Statistical analysis using OR with 95% CI demonstrated an overall significant difference with an OR score of 0.062 (0.023–0.163, p < 0.001). The OR score for dairy cattle was 0.119 (0.023–0.608, p = 0.006) and that for beef cattle was 0.154 (0.040–0.593, p = 0.003), respectively. Another risk factor was the water source, which was statistically significant for all cattle (p < 0.001). Our results indicate that natural pond water poses the highest risk compared with other water sources. There was no significant difference when analyzing each type of cattle, with a p = 0.681 for beef cattle and 1.000 for dairy cattle since no dairy cattle were fed with water from natural ponds. Statistical analysis of the effect of housing floors showed the same trend as that of the water source: It was statistically significant overall (p < 0.001), but insignificant for each type of cattle (p = 0.198 for dairy cattle and p = 0.123 for beef cattle). However, soil floors have a higher risk of infection than mixed concrete and soil floors. Finally, the farming system is another important risk factor. The risk of infection was higher in open farming systems than in closed systems, especially for beef cattle. The corresponding OR values (95% CI) for beef cattle were 0.135 (0.070–0.262, p < 0.001) overall and 0.252 (0.101–0.628, p = 0.002). In the case of dairy cattle, there was no significant relationship between the farming system and infection rates with an OR value of 1.316 (0.152–11.416, p = 1.000).

**Table 4:** Associated risk factor analysis for gastrointestinal nematode infection of dairy and beef cattle in Pak Chong district, Nakhon Ratchasima province.

Risk factors	Dairy cattle		Beef cattle		Overall	
		
	Positive n (%)	Negative n (%)	Positive n (%)	Negative n (%)	Positive n (%)	Negative n (%)
Age interval (years)						
0–1	4/12 (33)	8/12 (67)	26/44 (59)	18/44 (41)	30/56 (54)	26/56 (46)
2–3	4/29 (14)	25/29 (86)	14/24 (58)	10/24 (42)	18/53 (34)	35/53 (66)
4–5	0/37 (0)	37/37 (100)	9/16 (56)	7/16 (44)	9/53 (17)	44/53 (83)
>5	1/23 (4)	22/23 (96)	9/16 (56)	7/16 (44)	10/39 (26)	29/39 (74)
Total	9/101 (9)	92/101 (91)	58/100 (58)	42/100 (42)	67/201 (33)	134/201 (67)
p-value	NA		Pearson Chi-square 0.974		Pearson Chi-square < 0.001	
Food						
Natural grasses	7/34 (21)	27/34 (79)	55/86 (64)	31/86 (36)	62/120 (52)	58/120 (48)
Mixed	2/67 (3)	65/67 (97)	3/14 (21)	11/14 (79)	5/81 (6)	76/81 (94)
Total	9/101 (9)	92/101 (91)	58/100 (58)	42/100 (42)	67/201 (33)	134/201 (67)
OR (95% CI)	0.119 (0.023–0.608)		0.154 (0.040–0.593)		0.062 (0.023–0.163)	
p-value	0.006		0.003		<0.001	
Water source						
Natural ponds	0/0 (0)	0/0 (0)	21/33 (64)	12/33 (36)	21/33 (64)	12/33 (36)
Tap water	4/44 (9)	40/44 (91)	30/53 (57)	23/53 (43)	34/97 (35)	63/97 (65)
Groundwater	5/57 (9)	52/57 (91)	7/14 (50)	7/14 (50)	12/71 (17)	59/71 (83)
Total	9/101 (9)	92/101 (91)	58/100 (58)	42/100 (42)	67/201 (33)	134/201 (67)
Pearson Chi-square p-value	1.000 (Tap*Groud)		0.681		<0.001	
Housing floor						
Soil	3/18 (17)	15/18 (83)	51/83 (61)	32/83 (39)	54/101 (53)	47/101 (47)
Mixed	6/83 (7)	77/83 (93)	7/17 (41)	10/17 (59)	13/100 (13)	87/100 (87)
Total	9/101 (9)	92/101 (91)	58/100 (58)	42/100 (42)	67/201 (33)	134/201 (67)
OR (95% CI)	0.390 (0.088–1.733)		0.439 (0.152–1.271)		0.130 (0.064–0.262)	
p-value	0.198		0.123		<0.001	
Farming system						
Open system	1/14 (7)	13/14 (93)	48/71 (68)	23/71 (32)	49/85 (58)	36/85 (42)
Closed system	8/87 (9)	79/87 (91)	10/29 (34)	19/29 (66)	18/116 (16)	98/116 (84)
Total	9/101 (9)	92/101 (91)	58/100 (58)	42/100 (42)	67/201 (3)	134/201 (97)
OR (95% CI)	1.316 (0.152–11.416)		0.252 (0.101–0.628)		0.135 (0.070–0.262)	
p-value	1.000		0.002		<0.001	

OR=Odds Radio, CI=Confidence interval

## Discussion

GI nematodes are major parasites of the digestive system of cattle. GI nematode infections are ubiquitous and lead to poor health because of the destruction of digestive tissues [6]. Infection rates have increased in this century due to climate change [26]. Several nematodes have been reported to be pathogenic in cattle and cause gastroenteritis [27]. It usually occurs as a combination of several species located in different gut regions inside the host [28]. *Haemonchus*, *Ostertagia*, and *Teladorsagia* are the major parasites in the abomasum, whereas TS, *Nematodirus*, *Bunostomum*, *Capillaria*, and *Cooperia* inhabit the small intestine [2, 29]. *Chabertia*, *Oesophagostomum*, and *Trichuris* are nematode parasites located in the large intestine [30, 31].

Our present study reports the epidemiology of GI nematodes in cattle farmed in Pak Chong district, Nakhon Ratchasima province, Thailand, particularly in economically important dairy and beef cattle. The study area was selected due to the economic importance of cattle farming in it [17]. Microscopic examination of feces showed that nematodes are the major parasites in this area, with an overall infection rate of 33.3%. These results were comparable to previous studies by Yuwajita et al. [11], Thanasuwan *et al*. [13], and Baltrušis *et al*. [20]. Strongyle species are the major cattle GI nematodes in this area. Molecular examination indicated that *Ostertagia ortertagi* was the major species, accounting for 82.0% of all identified strongyle nematodes, followed by HC and TS However, only 52 strongyle-positive samples were found to have positive results with the primer sets used in this study (Table-2). A possible reason is that these samples might have been infected with other strongyle nematodes, i.e., *Cooperia* spp. and *Oesophagostomum* spp. [12]. This issue should be further investigated. Our results suggest that the prevalence of GI nematode infection is high, particularly in beef cattle. When compared with studies from other areas of the world, the results were a little different, but several factors could be involved [30, 32]. The beef cattle we studied mostly live freely without a strict farming system, which probably increases the likelihood of infective stage contact. *O. ortertagi*, the major species found in our study, demonstrated two different evolutionary groups. The smaller strain was related to the reference strain, whereas the larger strain was distinct. Different variants or other species belonging to the same genus may exist, but further studies are needed.

In addition to the genetic and molecular diversities, the associated risk factors were also analyzed. We found that young age, consumption of natural grass, consumption of natural pond water, housing floor, and farming system were associated with a high infection risk. Some factors, such as growth, grass consumption, and farmers’ behavior, are related to previous studies by Filipe *et al*. [26] and Vande *et al*. [33]. Interestingly, food and water sources were the main risk factors. Consumption of potentially infectious food, such as natural grasses and pond water, can increase the risk of infection, so farmers should avoid these sources as much as possible. Similarly, a housing floor consisting exclusively of soil, as well as an open farming system, could be a risk factor due to the diversity of nematodes that can enter the host by skin penetration. Therefore, it is possible to encourage farmers to manage their farms as closed systems with concrete or other safe floors.

## Conclusion

This study reports the epidemiology of GI nematodes in dairy and beef cattle in a large farming area of Thailand. The GI nematode infection is the major problem in this area, especially in beef cattle. These results could benefit farmers by lowering the GI nematode infection rates of their cattle. However, other farming areas should be investigated to represent the overall infection in Thailand and to increase the economic value of the country.

## Authors’ Contributions

AGK and CW: Conceptualized the study. CW, MJ, WP, and SR: Conducted the experiments, laboratory works, data collection. CW, AGK, PM, MJ, SC, WP, SR, RJ, WPh, and PP: Sample collection. PP, CW, PM, and AGK: Performed data analysis. RG, DK, and PC: Supervised the study. AGK, PM, and CW: Wrote the original draft. AGK, PM, and DK: Reviewed and edited the manuscript. All authors have read, reviewed, and approved the final manuscript.
